# Examining the Factorial Structure of the *Maternal Separation Anxiety Scale* in a Portuguese Sample

**DOI:** 10.3389/fpsyg.2020.571734

**Published:** 2021-01-13

**Authors:** Maryse Guedes, Lígia Monteiro, António J. Santos, Nuno Torres, Manuela Veríssimo

**Affiliations:** ^1^William James Center for Research – ISPA, Instituto Universitário, Lisbon, Portugal; ^2^Instituto Universitário de Lisboa (ISCTE-IUL), CIS-IUL, Lisbon, Portugal

**Keywords:** maternal separation anxiety, perceptions about separation effects on the child, employment-related separation concerns, child age, factorial structure

## Abstract

The increase in women’s labor market participation emphasizes the importance of understanding maternal separation anxiety, that is, the unpleasant maternal emotional state, due to the actual or anticipated short-term separation from the child. Drawing on the insights of the attachment and psychoanalytic perspectives, the *Maternal Separation Anxiety Scale* (MSAS) was developed to overcome existing measurement gaps. However, prior research did not replicate its original three-factor structure in the contemporary context and in other cultural settings, using large samples composed of mothers of preschool children. This study aimed to examine the factorial structure of the MSAS in a sample of 597 Portuguese mothers of children aged 5–84 months who completed the questionnaire. The exploratory factor analysis (EFA) conducted in subsample 1 revealed a four-factor structure: Maternal Negative Feelings, Beliefs about Exclusive Maternal Care, Need of Proximity, and Perceptions of Separation Benefits for Children. Confirmatory factor analyses conducted in subsample 2 revealed that the original three-factor structure revealed a poor fit, whereas the four-factor solution (obtained in the EFA) revealed an acceptable fit. As in previous studies, our findings report deviations from the original three-factor structure of the MSAS. Three of the newly identified factors seem to reflect specific sub-dimensions that originally guided item development in the MSAS, namely, maternal negative feelings, maternal attitudes about the value of exclusive maternal care, and the need of proximity with the child. The last factor appears to represent a refinement of original items pertaining to perceptions about separation effects for children.

## Introduction

In the last decades, women’s participation in the labor market is an increasing trend in the majority of economically developed countries (Organization for Economic Co-operation and Development, 2011). Due to these societal changes, there has been an increasing interest in the understanding of short-term mother–child separations (Robin, 2009). Almost 40 years ago (in the 1980s), several studies have highlighted the need to go beyond the focus on the consequences of short-term separations for children and furthermore to examine the maternal perspective (Hock, 1978, 1980), namely, maternal separation anxiety.

According to Hock et al. (1989), maternal separation anxiety can be defined as an unpleasant emotional state, due to the actual or anticipated short-term separation from the child. At moderate levels, this unpleasant emotional state is normative, especially during the first 6 months after childbirth (Hock et al., 1989), and decreases gradually as the child develops from infancy to school age (Hock and Schirtzinger, 1992). However, when maternal separation anxiety is excessively high, the mother exerts a rigid control on the mother–child relationship, undermining child’s autonomy and differentiation (Hock and Schirtzinger, 1989, 1992). Research has shown that heightened levels of maternal separation anxiety are associated with increased maternal anxiety and depression symptoms, lower parenting self-efficacy, overprotective parenting behaviors (Hock and Schirtzinger, 1992; Lutz and Hock, 2001; Hsu, 2004), and, ultimately, impaired child socioemotional competence during toddlerhood (Cooklin et al., 2013) and the preschool years (Veríssimo et al., 2003; Pessoa-Costa et al., 2015). Conversely, when maternal separation anxiety is excessively low, the mother does not seem to be properly connected to her child, enhancing child’s individuation too prematurely (Hock and Schirtzinger, 1989, 1992). Assessing maternal separation anxiety beyond infancy, using reliable and valid measures, is crucial to identify children who may be at increased risk of adverse developmental outcomes and to early intervene with them and their families.

### Theoretical and Empirical Underpinnings of Maternal Separation Anxiety

The interest on maternal separation anxiety, as a complex, multi-dimensional, and multi-determined construct, has been rooted in attachment and psychoanalytic perspectives that have shed light on mother’s biological and psychological need for proximity to her child (McBride and Belsky, 1988; Hock and Schirtzinger, 1992).

Bowlby (1973) established that maternal feelings toward mother–child separation can be understood as a result of a genetically determined predisposition to maintain physical proximity with her child, providing her protection against harm, comfort, and a secure base for exploration (Hock et al., 1989; Hock and Schirtzinger, 1992). Maternal separation anxiety emerges, when separation interferes with the mother’s ability to provide care and support (Hock and Schirtzinger, 1989). Complementarily to this perspective, Benedek (1970) acknowledged that the maternal personality structure influences the mother’s ability to remain physically and psychologically available to provide protection and security to her child, but also to give her the opportunities for healthy individuation–separation (Hock and Schirtzinger, 1989). Highly anxious mothers display an excessive need for proximity with their children, thus undermining child’s exploration in a context of emotional support and protection (Hock and Schirtzinger, 1992).

Hock’s research, based on laboratory observations and interviews (1978, 1980), has also emphasized that social and cultural values and norms about the acceptability of short-term mother–child separation and their benefits or risks for child development should also be considered. These norms can influence maternal perceptions about the separation effects on child, maternal concerns about employment-related separation, and, ultimately, maternal separation anxiety (Hock and Schirtzinger, 1989; Hock et al., 1989).

### The Maternal Separation Anxiety Scale (MSAS)

Drawing on extant theoretical and empirical data, Hock et al. (1989) developed the MSAS to overcome existing gaps in the measurement of maternal separation anxiety, by making available a reliable and valid self-report questionnaire that is not limited to a situational assessment of maternal anxiety, and is less time-consuming than laboratory observations and interviews. The exploratory factor analysis (EFA) conducted by Hock et al. (1989) in a sample of 623 American first-time postpartum mothers led to a final version of the MSAS, organized in three factors. The first factor (Maternal Separation Anxiety) assesses maternal feelings of sadness, worry, and/or guilt when separated from the child; beliefs about the importance of exclusive maternal care; and concerns about child’s ability to adapt to non-maternal care. The second factor (Perceptions about Separation Effects on the Child) measures maternal perceptions about child reactions to separation episodes and their positive effects to enhance child’s autonomy and sociability. The last factor (Employment-related Separation Concerns) assesses mother’s concerns about separation episodes to work outside home, depending on her interest in both professional career and parenting. The low magnitude of inter-factor correlations revealed that the three factors of MSAS were independent from each other’s, measuring different features of maternal separation anxiety.

Although the MSAS has been widely used worldwide, very few studies have examined the replicability of its original factorial structure in the contemporary context and in other cultures. From a theoretical standpoint, Hock et al. (1989) established that maternal separation anxiety is a multi-determined construct that can be influenced by sociocultural values and norms. Labor market participation has increasingly become a normal part of life for contemporary women in most economically developed countries (Organization for Economic Co-operation and Development, 2011), so that changes in maternal acceptability toward employment-related mother–child separations and on their perceived benefits or risks for child development need to be investigated in the present-day context.

From an empirical standpoint, the factorial structure of the MSAS was only preliminarily explored in France (Robin et al., 1999) and Romania (Costea-Bărluţiu, 2010). Both of these preliminary studies did not replicate Hock et al. (1989) original factorial structure. Robin et al. (1999) conducted an EFA in a sample of 60 first-time French mothers of children aged 18–30 months (mean age = 25 months), using the 35 items of the MSAS. Based on this exploratory analysis of items, the authors concluded that the factorial structure of the MSAS for the subscales Perceptions about Separation Effects on the Child (Factor 2) and Employment-related Separation Concerns (Factor 3) was not apparent in a sufficiently clear way. Therefore, Robin et al. (1999) decided to retain only one reliable factor (α = 0.90) assessing maternal separation anxiety, composed of 19 items extracted from the first (17 items) and third (two items) original factors of the MSAS. More recently, Costea-Bărluţiu (2010) conducted a confirmatory factor analysis (CFA) to test the original factorial structure of the MSAS. Based on a sample of 75 Romanian mothers of children aged 2–27 months (mean age = 8.40 months), the author found that the model fit deviated significantly from the theoretical goodness-of-fit indexes.

### The Present Study

Due to the potential influence of social norms in maternal separation anxiety (Hock et al., 1989) and the inconsistency of available empirical findings with Hock et al. (1989) original model, new studies need to examine the factorial structure of the MSAS in the present-day context and different cultural settings, using larger samples and robust exploratory factorial procedures. Furthermore, the attachment (Bowlby, 1973) and psychoanalytic (Benedek, 1970) perspectives establish that maternal separation anxiety is normative and healthy during infancy and typically decreases during the preschool years, as the child acquires new motor, cognitive, social, and emotional skills and becomes more independent from her mother (Hock et al., 1984; Hock and Schirtzinger, 1992). High levels of separation anxiety in mothers of preschool children do not allow children to engage in age-appropriate autonomous behaviors (Hock and Schirtzinger, 1992) and can impact negatively on their socioemotional competence (Veríssimo et al., 2003; Cooklin et al., 2013; Pessoa-Costa et al., 2015). The importance of a reliable and valid assessment of maladaptive levels of maternal separation anxiety beyond infancy justifies the need to explore the factorial structure of the MSAS in samples that include mothers of preschool children.

Thus, the present study aims to examine the factorial structure of the MSAS in a Portuguese sample, composed of mothers of preschool children. Based on the few available literature, we hypothesize that Hock et al. (1989) original three-factor model will not fit the data.

## Method

### Participants

The sample consisted of 597 mothers aged 19–48 years (*M* = 33.83, *SD* = 5.08); their education ranged from 1 year of education to university (*M*_*years*_ = 12.59 years, *SD* = 3.80). Most mothers were married (64%) and employed (84%), and worked at a mean of 6.56 h per day (*SD* = 2.95). Table 1 summarizes mother’s distribution with respect to age, education, and employment. Children’s ages ranged from 5 to 84 months (*M* = 55.06 months, *SD* = 12.56), and 51% were girls. Table 2 displays children’s age distribution with respect to gender. Children started to attend non-maternal care between 4 and 69 months (*M* = 24.79, *SD* = 16.18), spending at the time of the study between 1 and 11 h per day (*M* = 7.77, *SD* = 1.37), in non-maternal care.

**TABLE 1:** Mothers’ distribution, according to age, education, and employment (*N* = 597).

	*n* (%)
**Mothers’ age group**	
19–29 years	114 (19)
29–39 years	408 (69)
40–49 years	71 (12)
**Mother’s education**	
Elementary school	168 (28)
High school	201 (34)
University	225 (38)
Mother’s employment	
Employed	451 (84)
Unemployed	87 (16)

*The discrepancy between the total number of mothers (*N* = 597) and mothers’ distribution, according to age, education, and employment, is due to missing values.*

**TABLE 2:** Children’s age distribution, according to gender (*N* = 597).

	Child sex		
	
	Male	Female	Total
Child age group	*n* (%)	*n* (%)	*n* (%)
Less than 36 months	16 (2)	22 (4)	38 (6)
36–47 months	72 (12)	54 (9)	126 (21)
48–59 months	95 (16)	110 (18)	205 (34)
60–71 months	84 (14)	103 (17)	187 (31)
72–84 months	23 (4)	18 (3)	41 (7)

### Procedures

This study is part of a wider research project, approved by the [ISPA’s] Ethics Committee and by the Ministry of Education (reference n° 0092300008). After obtaining the approval of the authors, the MSAS was translated to Portuguese by two independent researchers, fluent in English, and was discussed to establish the Portuguese version of the MSAS. Then, the Portuguese version was back-translated to English by a third independent researcher, also fluent in English. The two English versions of the MSAS were compared to ensure the inexistence of significant differences on items’ meaning.

After the translation of the MSAS, the study was presented to preschools’ boards to obtain the necessary authorizations for data collection. From a total of 63 schools, 30 consented to participate, and 45 classes contributed to the study. Informed consents explaining the study aims, procedures, and exclusion criteria (child’s diagnosis of developmental delays) were sent to mothers of children aged 5–84 months. Eligible mothers who agreed to participate in the study signed the informed consent. After obtaining informed consents, preschool teachers sent the self-report questionnaires to mothers to be completed at home. After completion, mothers returned the self-report questionnaires to preschool teachers in a closed envelope. Forty-one percent of the questionnaires were returned with all the information.

### Instruments

#### Sociodemographic Questionnaire

In this questionnaire, mothers were asked to provide sociodemographic information (age, marital status, years of education, employment status, child age, and sex).

#### MSAS (Hock et al., 1989)

This self-report questionnaire consists of 35 items, answered in a Likert scale ranging from 1 (*Strongly Disagree*) to 5 (*Strongly Agree*) and organized in three factors. The composition and meaning of each factor were fully described in Section “Introduction.” Punctuations of each factor item are averaged to yield a factor score. Higher scores in Maternal Separation Anxiety (Factor 1) indicate more maternal worry, sadness, and guilt; increased desire of proximity; and stronger beliefs about the superiority of exclusive maternal care. Higher scores in Perceptions about Separation Effects on the Child (Factor 2) represent stronger beliefs that the child does not benefit from separation experiences. Higher scores in Employment-related Separation Concerns (Factor 3) represent higher maternal concerns about separating from the child to work outside home. In the original study, Cronbach’s alphas for each factor were 0.90 (Factor 1), 0.71 (Factor 2), and 0.78 (Factor 3). The punctuations of the 35 items are also averaged to yield a global score. Higher global scores reflect increased maternal separation anxiety, perceptions of negative separation effects on the child, and employment-related separation concerns. In the original study, Cronbach’s alpha for the global score was 0.88.

### Data Analysis

Data analyses were conducted using IBM SPSS 25.0 and AMOS 25.0. Missing data were excluded. The exploration of Mahalanobis distances (*D*^2^, *p*1 < 0.001, *p*2 < 0.001) led to the removal of 96 subjects (identified as multivariate outliers). Absolute values of skewness (Sk < |3|, ranging from -1.54 to 1.14) and kurtosis (Ku > |7|, ranging from -0.93 to 3.46) did not indicate a violation of normal distribution (Byrne, 2010).

A split-half method was used for cross-validation purposes. Specifically, the entire study sample was randomly divided into two halves (subsample 1: *n* = 304; subsample 2: *n* = 293). Due to the potential influence of social norms in maternal separation anxiety (Hock et al., 1989), the inconsistency of available empirical findings with Hock et al. (1989) original model, and the aim of examining the factorial structure of the MSAS in a sample composed by mothers of preschool children, an EFA using principal axis factoring method with Promax rotation was conducted in subsample 1 to explore the factor structure of the MSAS. Given that it has been recognized as one of the most accurate approach, parallel analysis (PA) was used to determine the number of factors to retain (O’Connor, 2000). Both pattern and structure matrixes of item factor loadings were examined to identify items that reflected theoretically meaningful and interpretable factors. Items were retained if loadings were ≥ 0.40 and the difference between cross-loading factor loadings was sufficiently large (i.e., at least 0.30; Henson and Roberts, 2006).

In subsample 2, an item-level CFA was performed to compare the model fit of Hock et al. (1989) original three-factor model (Model 1), Robin et al. (1999) one-factor model, and the EFA’s model (Model 3). The method of estimation was maximum likelihood and bootstrap procedures were applied. Goodness of fit was verified using multiple fit indices: comparative fit index (CFI), Tucker–Lewis index (TLI), root-mean-square error of approximation [RMSEA; 90% confidence interval (CI)], and standardized root-mean-squared residual (SRMR). According to Byrne (2010), CFI > 0.90, TLI > 0.95, RMSEA < 0.08, and SRMR ≤ 0.08 indicate an acceptable fit. Furthermore, the χ^2^/degrees of freedom ratio (χ^2^/df) was examined, considering that values between 2 and 5 indicate an acceptable fit (Byrne, 2010). Akaike information criterion (AIC; Bozdogan, 1987) and the modified expected cross-validation index (MECVI; Browne and Cudeck, 1989) were used to estimate improvements across alternative models, with lower values being indicative of a best fitted model. Following the standards of Hair et al. (2010), item individual reliability (λ ≥ 0.50), internal consistency (λ^2^ ≥ 0.25), and composite reliability of all factors (≥0.70) were examined.

## Results

### Exploratory Factor Analyses

The EFA yielded nine factors with eigenvalues greater than 1. However, PA and theoretical interpretation suggested the retention of four interpretable and meaningful factors. Based on the inspection of pattern and structure matrixes of item factor loadings, items with loadings lower than 0.40 and multiple loadings (in which the difference between cross-loadings were lower than 0.30) that did not reflect theoretically meaningful factors were removed. This set of criteria resulted in the removal of 19 items. Nine of the removed items pertained to the first original factor (Maternal Separation Anxiety) and focused on maternal concerns about the ability of the child to adapt to non-maternal care (e.g., “children will be afraid in a new place without their mother”) and the ability of non-maternal caregivers to answer child’s emotional and physical needs [e.g., “when I am away from my child, I often wonder if his/her physical needs (dry diapers, enough to eat, etc.) are being met”]. Three of the removed items pertained to the second original factor (Perceptions of Separation Effects for Children) and focused on maternal perceptions about child reactions to separation episodes (e.g., “if a child is independent and outgoing, he/she will make friends easily without his/her mother’s help). Finally, all the seven items pertaining to the third original factor (Employment-related Separation Concerns) were removed, that is, items focusing on mother’s concerns about separation episodes to work outside home, depending on her interest in both professional career (e.g., “my life wouldn’t be complete without a career”) and parenting (e.g., “I would not regret postponing my career in order to stay home with my child”). Sixteen items were retained. The final EFA solution on the retained items is presented in Table 3.

**TABLE 3:** Exploratory factor analysis of the *Maternal Separation Anxiety Scale* in subsample 1 (*n* = 304).

	F1	F2	F3	F4
% of explained variance	**27%**	**12%**	**8%**	**8%**
27. When I am separated from my child, I wonder whether he/she is crying and missing me.	**0.78**	-0.02	-0.18	0.08
29. I worry that my child is never completely comfortable in an unfamiliar setting if am not with him/her.	**0.78**	-0.05	0.04	0.07
28. I don’t enjoy myself when I’m away from my child.	**0.71**	-0.08	0.06	0.04
24. My child is afraid and sad when he/she is not with me.	**0.54**	0.19	0.04	-0.06
32. I worry when someone else cares for my child.	**0.49**	0.20	-0.01	0.01
18. I am naturally better at keeping my child safe than any other person.	0.09	**0.67**	-0.02	-0.04
8. I am more concerned with my child’s physical safety than a babysitter or teacher.	0.00	**0.66**	0.03	-0.04
02. My child is happier with me than with babysitters or teachers.	-0.17	**0.57**	-0.12	0.18
23. My child prefers to be with me more than with anyone else.	0.18	**0.44**	0.07	0.13
26. My child needs to spend time away from me in order to develop a sense of being an individual in his/her own right.	-0.18	-0.04	**0.72**	0.09
16. It is good for my child to spend time away from me so that he/she can learn to deal independently with unfamiliar people and new situations.	-0.06	0.02	**0.65**	0.08
31. Exposure to many different people is good for my child.	0.18	-0.02	**0.51**	-0.26
34. There are times in the lives of young children when they need to be with people other than their mothers.	0.10	-0.06	**0.44**	0.11
7. Holding and cuddling my child makes me feel so good that I really miss the physical closeness when I’m away.	0.17	-0.02	0.07	**0.65**
17. I like to have my child close to me most of the time.	0.06	-0.10	0.06	**0.59**
1. I miss holding or cuddling my child when I am away from him/her.	-0.05	0.17	-0.01	**0.54**

*Exploratory factor analysis, using principal axis factoring method with Promax rotation. F1 refers to Factor 1 (Maternal Negative Feelings). F2 refers to Factor 2 (Need of Proximity). F3 refers to Factor 3 (Beliefs about Exclusive Maternal Care). F4 refers to Factor 4 (Separation Benefits for Children).*

Factor 1 consists of five items, explaining 27% of the variance, pertaining to Maternal Negative Feelings. Factor 2 was composed of four items, explaining 12% of the variance, pertaining to Beliefs about Exclusive Maternal Care. Factor 3 was composed of four items, explaining 8% of the variance, pertaining to Perceptions of Separation Benefits for Children. Factor 4 consisted of three items, explaining 8% of the variance, pertaining to Need of Proximity.

### Confirmatory Factor Analyses

Table 4 presents the findings of confirmatory factor analyses, comparing Hock et al. (1989) original three-factor model (Model 1), Robin’s one-factor model (Model 2), and EFA’s four-factor model (Model 3).

**TABLE 4:** Goodness-of-fit indexes of confirmatory factor analyses in subsample 2 (*n* = 293) comparing Hock et al. (1989) original three-factor model, Robin et al. (1999) one-factor model, and EFA’s four-factor model.

	χ^2^/g.l	CFI	TLI	SRMR	RMSEA (90% CI)	AIC	MECVI
Model 1—Hock et al. (1989) original three-factor model	2.76	0.67	0.65	0.09	0.078 [0.073–0.082]	1750.80	6.10
Model 2—Robin et al. (1999) one-factor model	3.74	0.73	0.70	0.08	0.097 [0.088–0.105]	644.65	2.23
Model 3—EFA’s model	2.11	0.91	0.92	0.06	0.06 [0.050–0.073]	282.32	0.983
Model 4—Second-order EFA’s model	2.11	0.91	0.89	0.06	0.06 [0.050–0.073]	282.63	0.983

As shown in Table 4, Model 1 (Hock’s original three-factor model) revealed a poor fit. The examination of item loadings revealed that 15 of the 35 items had values lower than 0.50: seven pertaining to Factor 1 (Maternal Separation Anxiety), describing the need of proximity and concerns that non-maternal caregivers do not adequately meet the physical and/or emotional needs of her child; three pertaining to Factor 2 (Perceptions of Separation Effects for Children), describing mother’s perceptions of her child’s reactions to actual separation episodes; and five pertaining to Factor 3 (Employment-related Separation Concerns) describing the importance of career in mother’s life. Furthermore, the examination of modification indexes (>11) evidenced the presence of several item cross-loadings (especially in Factors 1 and 2) and several covariances between errors, supporting that the model does not fit the data.

Table 4 also shows that Model 2 (Robin’s one-factor model) revealed a poor fit. Although AIC and MECVI indexes were lower than in Model 1, the examination of item loadings revealed that six of the 19 items had values lower than 0.50: two items describing mother’s desire to have more time for career and to postpone career to stay at home with the child, two items describing the need of proximity, and two items focusing on concerns about child’s ability to adapt to non-maternal caregivers. Furthermore, the examination of modification indexes (>11) evidences the presence of several covariances between errors, supporting that the model does not fit the data.

In contrast, Model 3 (EFA’s four-factor model) revealed acceptable fit. AIC and MECVI values were lower than in Models 1 and 2 (see Table 4).

**FIGURE 1:**
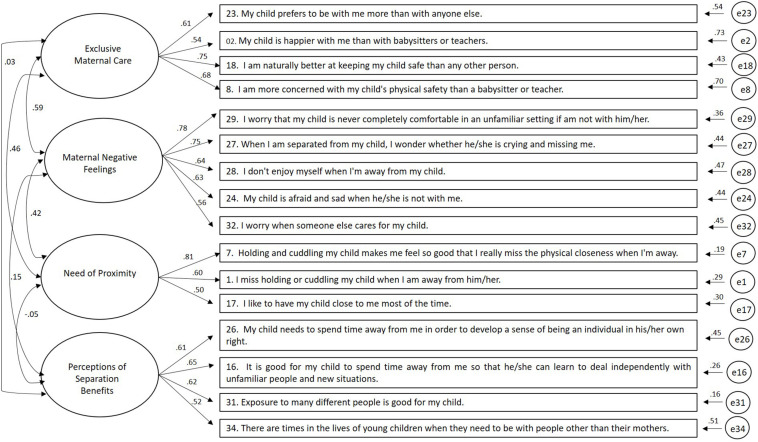
Standardized parameters of the four-factor model of the *Maternal Separation Anxiety Scale*.

Figure 1 presents the standardized parameters of Model 3. All items had adequate individual reliability (l ≥ 0.50, ranging from 0.51 to 0.81) and internal consistency (l^2^ ≥ 0.25, ranging from 0.25 to 0.65). The composite reliability of each factor ranged from 0.70 (Factor 4—Need of Proximity) to 0.81 (Maternal Negative Feelings).

**FIGURE 2:**
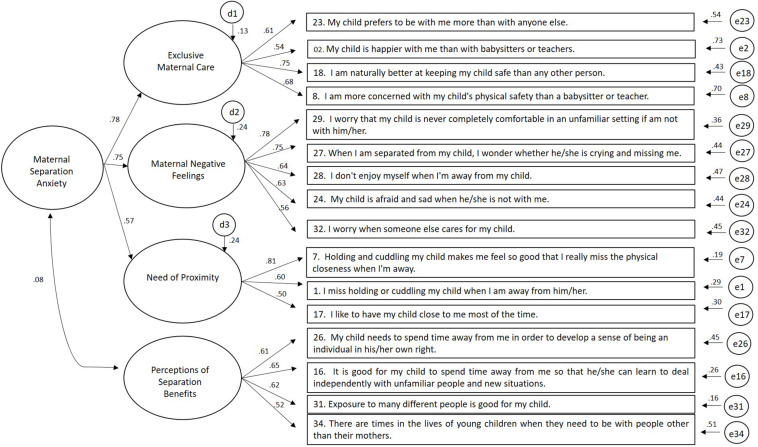
Standardized parameters of the second-order model of the *Maternal Separation Anxiety Scale*.

Factors 1, 2, and 4 of the EFA’s four-factor model (Model 3) represent sub-dimensions that were conceptualized as part of a broader dimension (Maternal Separation Anxiety) by Hock et al. (1989). Consequently, a second-order factor (Maternal Separation Anxiety) with three associated first-order factors (Maternal Negative Feelings, Beliefs about Exclusive Maternal Care, Need of Proximity) was introduced in the model (Model 4). Table 4 shows that Model 4 (EFA’s second-order model) revealed an acceptable fit but did not represent an improvement when compared with the first-order EFA’s four-factor model (Model 3). Figure 2 presents the standardized parameters of Model 4. The reliabilities of the first-order dimensions that constitute the higher-order factor (Maternal Separation Anxiety) were 0.57 (Need of Proximity), 0.75 (Maternal Negative Feelings), and 0.78 (Beliefs about Exclusive Maternal Care).

## Discussion

This study aimed to investigate the factorial structure of the MSAS (Hock et al., 1989) in a Portuguese sample, composed of mothers of preschool children. Examining the factorial structure of the MSAS is crucial, due to the potentialities of its application in clinical and educational settings to assess maladaptive levels of maternal separation anxiety and early intervene with families. In fact, extreme levels of maternal separation anxiety can contribute to dysfunctional mother–child relationships and overprotective parenting behaviors that interfere with the development of child’s autonomy (Hock and Schirtzinger, 1992; Peleg et al., 2006). Similarly, the absence of maternal separation anxiety can undermine mother’s emotional connection to her child (Peleg et al., 2006) and results in premature child’s individuation (Hock and Schirtzinger, 1989, 1992).

In line with our hypothesis, Hock’s original three-factor model (i.e., Maternal Separation Anxiety, Perceptions of Separation Effects on Children, Employment-Related Concerns) did not fit the data. Our findings, as in previous studies (Robin et al., 1999; Costea-Bărluţiu, 2010), report deviations from the original factorial structure, using a larger and a sample of mothers of preschool children.

Exploratory and confirmatory factor analyses suggested the adequacy of a four-factor model. Three of the newly identified factors seem to reflect part of the specific sub-dimensions that originally guided item development for the Maternal Separation Anxiety Factor of the MSAS (Hock et al., 1989), namely, the expression of maternal negative feelings (i.e., sadness, worry, apprehension, and guilt) during actual or anticipated short-term separation episodes from the child (Factor 1), maternal attitudes about the value and importance of exclusive maternal care (Factor 2), and the need of proximity with the child (Factor 4). Although it did not represent an improvement, the acceptable fit of the second-order model that comprised a higher-order factor (Maternal Separation Anxiety) with three associated first-order factors (Maternal Negative Feelings, Beliefs about Exclusive Maternal Care, Need of Proximity) seems to support this interpretation. These three factors comprise 12 of the 21 items pertaining to Hock’s Maternal Separation Anxiety factor (Hock et al., 1989). Child mean age at the time of data collection (i.e., 55 months) and child age when starting childcare attendance (i.e., 25 months) in our sample may have contributed for the removal of items focusing on maternal concerns about the ability of the child to adapt to non-maternal care and the ability of non-maternal caregivers to answer child’s emotional and physical needs. Retained items appeared to reorganize themselves to encompass the core features of maternal separation anxiety that have been conceptualized in the attachment (Bowlby, 1973) and psychoanalytic (Benedek, 1970) frameworks. Whereas the first factor (Maternal Negative Feelings) seems to reflect mother’s unpleasant emotional state, due to the actual or anticipated short-term separation from the child (Hock et al., 1989), the second factor (Beliefs related to Exclusive Maternal Care) appears to be more directly associated with features of mother’s personality structure described by Benedek (1970). In fact, Beliefs related to Exclusive Maternal Care encompasses maternal attitudes focusing on the superiority of maternal caregiving when compared with non-maternal care, which, in turn, can limit mother’s ability to be available to protect her child and simultaneously provide him/her opportunities for healthy individuation–separation. In accordance with the attachment framework (Bowlby, 1973), Need of Proximity (Factor 4) appears to reflect an excessive need of closeness with the child that can undermine child’s exploration in a context of emotional support and protection. The moderate inter-correlations between Factors 1, 2, and 4 support the idea that Maternal Negative Feelings, Beliefs related to Exclusive Maternal Care, and Need of Proximity seem to assess different, but correlated features of the same construct—i.e., maternal separation anxiety.

Contrary to Factors 1, 2, and 4, the remaining factor seems to be more directly associated with social and cultural values that have been considered to affect maternal separation anxiety, namely, on the acceptability of short-term mother–child separations and their perceived benefits or risks for child development (Hock, 1978, 1980; Hock and Schirtzinger, 1989; Hock et al., 1989). More specifically, Factor 3 seems to represent a refinement of items pertaining to Hock’s Perceptions about Separation Effects for Children (Hock et al., 1989). This reorganization is consistent with the findings obtained in the EFA conducted by Robin et al. (1999) in a French sample, showing that Hock’s Perception about Separation Effects on the Child and Employment-Related Separation Concerns factors was not apparent in a clear way.

In our study, Factor 3 (Perceptions of Separation Benefits for Children) includes only items describing the child’s ability to profit from non-maternal care, with a particular emphasis on its advantages for the promotion of child social skills. Furthermore, Hock et al. (1989) Employment-related Separation Concerns factor was not retained in our factorial model. These findings may reflect the influence of the social changes in women’s role in labor market participation in most economically developed countries (Organization for Economic Co-operation and Development, 2011) that make the utilization of non-maternal care services a normal part of life for contemporary families. In fact, Organization for Economic Co-operation and Development (2016) statistics have shown an increase in the number of women who are in paid work during the childbearing years and in the participation rates (around or above 70%) for children aged 3–5 years in early childhood education and care services in countries across the OECD and Europe during the last decades. The sociodemographic characteristics of mothers in our sample (84% of employed mothers) and the average number of hours that children spend in non-maternal care per day (7.78 h) are consistent with these OECD statistics. This may explain why items focusing on child’s ability to adapt to non-maternal care, the desire to have more time for career, and the availability to renounce or postpone career to stay at home with children were perceived as less meaningful for mothers in our sample.

Although its composition was modified, when compared with Hock’s original model (1989), Factor 3 displayed low-magnitude inter-correlations with the remaining newly identified factors. This is consistent with the idea of Hock et al. (1989) to provide a self-report questionnaire measuring factors that are independent from each other’s, in order to capture the multiple determinants (i.e., instinctive base, mother’s personality structure, cultural standards on the acceptability of separation, and its benefits for child development) that can influence maternal separation anxiety.

To the best of our knowledge, this is the first study to examine the factorial structure of the MSAS in the contemporary context and a new cultural setting, using both exploratory and confirmatory factor analyses in a larger sample, composed of mothers of preschool children. However, some limitations need to be acknowledged. First, this study was based on a convenience sampling method and the sample mainly consisted of employed mothers, aged 30–39 years, with at least high school education. The composition of the sample was in line with the national and European statistics concerning women’s mean age at motherhood at the time of data collection (Fundação Francisco Manuel dos Santos, 2020a). However, the sampling method and the higher proportion of women who were employed and had at least high school education when compared with available statistics at the time of study (Fundação Francisco Manuel dos Santos, 2020b,c) limit the generalizability of the findings. With respect to children’s characteristics, our sample under-represented children aged less than 36 months and children aged 71–84 months, thus limiting the assessment of measurement invariance regarding child age group. Second, exploratory and confirmatory factor analyses were conducted in two separated subsamples for cross-validation purposes. However, the replicability of the newly identified four-factor model and its measurement invariance was not tested in a large independent sample. The study used the most commonly recommended approach for multivariate outlier detection in a variety of contexts and fields (Finch, 2012). In spite of its usefulness, this approach has also weaknesses that may justify the use of alternative methods (Finch, 2012) based on robust estimators in future research. Lastly, the concurrent validity of the MSAS was not assessed in the present study.

Future studies are needed to test the factorial structure of the MSAS in large independent samples. Furthermore, future studies need to investigate the measurement invariance of the MSAS across child (e.g., age and sex) and/or maternal (e.g., employment status and education) characteristics and across cultures. Examining measurement invariance across cultures may be particularly useful to clarify whether items focusing on sociocultural factors (i.e., acceptability of mother–child separations due to employment and its benefits for child development) that can influence maternal separation anxiety are interpreted by mothers in a similar way.

## Data Availability Statement

The raw data supporting the conclusions of this article will be made available by the authors, without undue reservation.

## Ethics Statement

The studies involving human participants were reviewed and approved by the ISPA Ethics Committee. Written informed consent to participate in this study was provided by the participants’ legal guardian/next of kin.

## Author Contributions

MV and AS contributed to conception of the work. LM and NT contributed to data collection. MG contributed to data analysis, data interpretation, and drafting the manuscript. MG, MV, and AS gave final approval of the version to be published and agreed to be accountable for all aspects of the work. All authors contributed to the article and approved the submitted version.

## Conflict of Interest

The authors declare that the research was conducted in the absence of any commercial or financial relationships that could be construed as a potential conflict of interest.
